# The Influence of Textile Type, Textile Weight, and Detergent Dosage on Microfiber Emissions from Top-Loading Washing Machines

**DOI:** 10.3390/toxics12030210

**Published:** 2024-03-12

**Authors:** Pongsiri Julapong, Palot Srichonphaisarn, Thidarat Meekoch, Carlito Baltazar Tabelin, Onchanok Juntarasakul, Theerayut Phengsaart

**Affiliations:** 1 Department of Mining and Petroleum Engineering, Faculty of Engineering, Chulalongkorn University, Bangkok 10330, Thailand; 2 Department of Mining and Materials Engineering, Faculty of Engineering, Prince of Songkla University, Songkhla 90110, Thailand; 3 Department of Materials and Resources Engineering and Technology, College of Engineering, Mindanao State University—Iligan Institute of Technology, Iligan City 9200, Philippines; 4 Resource Processing and Technology Center, Research Institute for Engineering and Innovative Technology (RIEIT), Mindanao State University—Iligan Institute of Technology, Iligan City 9200, Philippines; 5 Applied Mineral and Petrology Research Unit (AMP RU), Department of Geology, Faculty of Science, Chulalongkorn University, Bangkok 10330, Thailand

**Keywords:** microplastics, laundry, fabric, detergents, water treatment

## Abstract

The use of washing machines to wash textiles gradually breaks down synthetic fibers like polyethylene terephthalate (PET) or polyester (PES) in diverse clothing materials, a process that is growing in notoriety because it generates microplastics (MPs). In this study, we investigated the emission of microfibers, including both microplastic fibers (MPFs) and natural fibers (MFs), from top-loading washing machines. Our investigation focused on four popular textiles with prevalent weave structures (plain, satin, and twill): (i) PES, (ii) tetron cotton (TC), (iii) chief value cotton (CVC), and (iv) cotton (CO) fabrics. This study also examined the effects of textile weight and detergent dosage on MF emissions. After washing, MFs were collected through filtration, and their concentrations were determined using micro-Fourier Transform Interferometry (μFTIR). The results showed varying concentrations of MFs in the washing effluent depending on the type of textile. Specifically, CVC exhibited the highest emission at 4022 particles/L, followed by TC, PES, and CO at 2844 particles/L, 2382 particles/L, and 2279 particles/L, respectively. The hydrophobic nature of PES makes this type of textile prone to rapid degradation in detergent-rich environments, leading to high MF emissions. Additionally, the mechanical properties of textiles, such as tensile and bending strengths, may play a crucial role in the generation of MFs in washing machines. Textiles made of CO with twill weaves demonstrated superior strength and correlated with lower emissions of MFs. In comparison, textiles made of CVC and satin weave exhibited lower mechanical properties, which could explain their high emissions of MFs. Finally, the MF emissions of textiles composed of PES and TC, which are plain weaved, could be attributed to their intermediate mechanical properties compared with those of CVC and CO.

## 1. Introduction

Microplastics (MPs) are small plastic particles ranging in size from 1 to 5000 μm. They have become a major environmental concern due to their widespread presence in various ecosystems. MPs are potentially harmful to organisms because they can cause oxidative stress, cytotoxicity, and can be easily translocated to other tissues [1,2,3,4]. The negative impacts of MPs to the environment, biota, and organisms are exacerbated by their small particle size, hydrophobic surface, and abundant organic surface functional groups. Their small size enables easy transport and migration through wind and water, while their hydrophobic nature and surface functional groups facilitate the adsorption of inorganic and organic pollutants. These properties make MPs ideal carriers of harmful microorganisms and potentially toxic chemicals in the environment [5]. Moreover, MPs have been found to carry lead, cadmium, chromium, barium, copper, cobalt, arsenic, aluminum, iron, manganese, and zinc, persistent chemical pollutants (PCPs) like phthalate esters (e.g., dimethyl phthalate (DMP), diethyl phthalate (DEP), dibutyl phthalate (DnBP), benzylbutyl phthalate (BBZP), di(2-ethylhexyl) phthalate (DEHP), and di-n-octyl phthalate (DnOP)), and organic pollutants (e.g., polycyclic aromatic hydrocarbons (PAHs), polychlorinated biphenyls (PCBs), and organochlorine pesticides (OCPs)) [6,7,8,9,10]. 

The release of MPs into the environment is influenced by various sources and anthropogenic activities. These include processes in various sectors such as manufacturing, energy generation, chemical production, and resource extraction. Additionally, plastics from improperly disposed of municipal solid and liquid wastes naturally degrade in the environment, releasing MPs. Generally, the occurrence of MPs is categorized into primary and secondary based on their origin. Primary MPs are directly released into the environment, such as microbeads found in many cosmetic products and microplastic fibers (MPFs) generated from washing clothing and textiles. In contrast, secondary MPs are by-products resulting from the natural weathering and degradation of larger plastic materials and wastes in the environment [11]. 

MPs originating from inland areas enter water bodies through various pathways, including domestic wastes, wastewater treatment plants (WWTPs), industrial effluents, surface run-offs, wind currents, and improper plastic disposal practices [12,13,14,15,16]. According to Meijer et al. [17], the Philippines is the top contributor among the top 10 countries generating ocean plastic wastes, estimated at 0.356 million metric tons (Mt). Following the Philippines are India (0.127 Mt), Malaysia (0.073 Mt), China (0.071 Mt), Indonesia (0.056 Mt), Brazil (0.038 Mt), Vietnam (0.028 Mt), Bangladesh (0.025 Mt), Thailand (0.023 Mt), and Nigeria (0.019 Mt). These findings highlight the significant role these nations play in mitigating the global ocean MP pollution problem. 

The 2030 United Nations (UN) agenda includes 17 Sustainable Development Goals (SDGs) designed to address fundamental sustainability issues faced by humanity and the planet. One of these goals, SDG 14 “Life below water”, focuses on the impacts of MP emissions on marine environments, reflecting the growing social awareness surrounding this issue. As a result, there has been a rapid increase in research related to MPs, not only in understanding their environmental and biological impacts but also the modes of their release into marine ecosystems. In fact, the number of publications on MPs and their emissions in Scopus has grown exponentially since 2016 [18], with 4309 articles published by the year 2023. This rapid increase in research highlights the significance and urgency of addressing the impacts of MP pollution on society and the environment.

Domestic wastewater streams, both treated (from wastewater treatment plants (WWTPs)) and untreated, are a significant source of MPs. Magni et al. [19], for example, reported that influents of WWTPs contain MPs ranging from 0.43 to 1030 particles/L, and their treatment could reduce the concentration of MPs to between 0.31 and 30.3 particles/L. However, conventional technologies employed by WWTPs have MP removal efficiencies as low as 60%, with an average of around 84% [19,20,21,22,23,24,25,26,27,28,29,30]. This means that approximately 16% of MPs are inevitably released into the environment from domestic wastewaters even after treatment. MP contamination also arises from the use of sludge as fertilizer in agriculture, contributing to the presence of MPs in water bodies [31]. Synthetic fibers in sludge, sludge products, and WWTP effluents have been discovered by the pioneering works of Habib et al. [32] and confirmed by Zubris et al. [33], highlighting the contribution of these materials to MP pollution. In Thailand, Tadsuwan and Babel [34] found that a representative WWTP influent contained an average of 77 ± 7.21 particles/L of MPs, which was reduced to an average of 10.67 ± 3.51 particles/L after treatment. These authors also highlighted that MPFs were the most dominant type of MPs found in WWTP influent, constituting over 60% of the total. Due to their very small size and fibrous morphology, MPFs are not effectively captured by conventional WWTPs, leading to their release into rivers and oceans [35]. Browne et al. [36] conducted pioneering research that clearly demonstrates the potential contribution of washing synthetic clothing to the accumulation of MPFs in marine environments.

A significant portion of MPFs found in domestic wastewater streams come from washing textiles made from natural and plastic or synthetic fibers. As the population has grown, the global production of synthetic and natural fibers for textiles has also increased to meet the demand. Textile manufacturing uses a variety of fiber types, including natural, synthetic, and blends of both, such as polyester–cotton (PES–CO). Synthetic fibers have become increasingly integrated into textiles over the past 50 years, alongside traditional materials like cotton (CO), wool, and linen, due to abundance and lower costs. Between 2000 and 2020, the demand for synthetic fibers nearly doubled from 57 Mt to 111 Mt, with projections suggesting it will reach 145 Mt by 2030 [18]. As of 2021, synthetic fibers dominate the global fiber production, accounting for approximately 64% of the market. Among the synthetic fibers, polyester (PES) or polyethylene terephthalate (PET) holds the majority share at 54%, followed by polyamide (PA) at 5%, while the remaining 5.2% comprises other fibers like polypropylene (PP), acrylics, and elastane [37].

MPFs have been found in different environmental media globally, including seawater, seafloor sediments, estuaries, wastewater treatment plants, shores, and various organisms [38]. According to a report by Boucher and Friot [39], MPFs account for 34–35% of the overall presence of MPs in marine environments. Belzagui et al. [40] estimated an annual influx of approximately 1.4 × 10^17^ MPFs into oceans, while Yang et al. [41] identified laundry wastewater, particularly from washing machines, as the primary source of MPFs. Because of these previous works, several commercially available devices have been developed to capture and reduce the release of microfibers (MFs; including both MPFs and natural MFs) from washing machines. These devices typically consist of filters that can be installed either internally or externally. One example is the “Cora ball”, which is designed to be placed alongside clothing in the washing machine. The ball incorporates stalks equipped with hooks specifically designed to capture microfibers, taking inspiration from the efficient filtering system observed in coral reefs. Other solutions include the “Guppyfriend washing bag” and “Fourth element washing bag,” both of which aim to reduce pilling and reduce fiber loss in washing machines. Additionally, there are external filters such as “XFiltra” and “Planet care” that can be installed along the effluent pipe of washing machines to capture MFs. Another relevant device in this context is the “Lint LUV-R”. These innovations collectively represent efforts to address and mitigate the release of MFs from washing machines [42]. However, it is important to note that these devices have a limited effectiveness in capturing MFs, with a capture rate of approximately 78%, leaving the remaining 22% to be released into the ecosystem [42].

To effectively mitigate the generation of MFs from household washing machines, it is important to understand the factors and mechanisms controlling textile degradation during washing. In this study, the effects of textile type and configuration, detergent concentration, and textile weight were investigated. Four commonly used textiles in clothing—CO, two ratios of PES–CO blends, and pure PES—were selected as representative samples. The textile samples were washed in a top-loading washing machine with detergent using various washing parameters. The initial washing machine effluent was then collected, filtered and the amount of MFs generated was quantified using micro-Fourier Transform Interferometry (μFTIR; sometimes called Fourier transform infrared microscopy (FTIR microscopy)). It is worth noting that the term “microfiber” is used in the textile industry to refer to fabrics made of fine PES or polyamine fibers. These fibers have measurements of less than 1 denier (mass in grams of 9000 m) and a fiber cross-section smaller than 10 μm [43] and should not be confused with MPFs. In this study, the term “microfibers (MFs)” is defined as the synthetic and natural fibers released from fabrics during washing with sizes of <5 mm. The results of this research will improve the current understanding of how MFs are generated in washing machines, including the key factors that influence MF release. This study will also provide valuable insights to policymakers, industry stakeholders, and the public in tackling MP pollution. 

## 2. Materials and Methods

**Figure 1:**
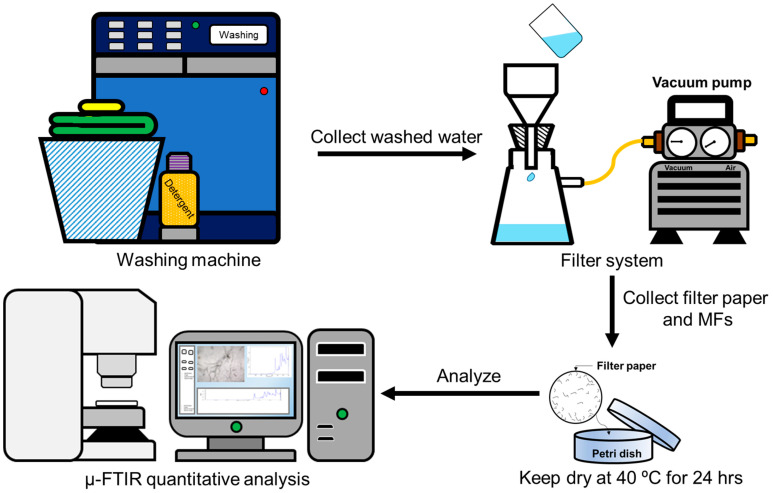
A schematic diagram of the experimental procedures used in this study.

The experimental procedures in this study are illustrated in Figure 1, including sample preparation, textile washing in a top-loading washing machine, washed water filtration using a 2.7 µm pore filter (with a diameter of 47 mm), and MF collection, capturing the filter images using µ-FTIR and performing manual counting for MF quantification and calculation. Previous works have highlighted notable discrepancies in the quantification of MFs during the laundry process, which are primarily attributed to inconsistencies in research methods and counting methodologies. From our review of the literature, it is evident that visual inspection with microscopes is the most commonly used approach for recognizing and measuring MFs because it is easy, straightforward, and reliable [44].

### 2.1. Samples

This study examined four distinct textile materials: (i) cotton (CO: 100% cotton), (ii) chief value cotton (CVC: 70% cotton and 30% polyester), (iii) tetron cotton (TC: 35% cotton and 65% polyester), and (iv) polyester (PES: 100% polyester). To ensure consistency and represent typical clothing products, all textile samples were cut into dimensions of 50 cm × 50 cm. For the washing experiments, a widely used and commercially available powder detergent in Thailand was purchased from a local supermarket. The detergent’s main components, as listed on the label, include anionic surfactant, zeolite, sodium carbonate, and sodium carboxymethyl cellulose.

### 2.2. Textile Washing Using Top-Loading Washing Machine

#### 2.2.1. Washing Step

**Figure 2:**
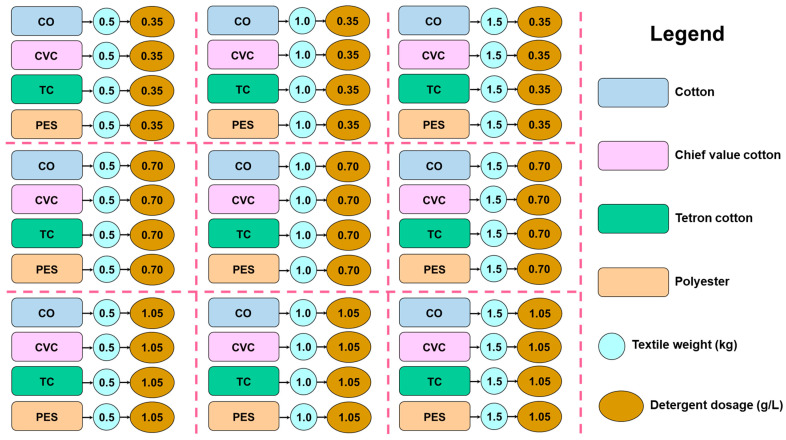
Parameters in the washing experiments of four types of textiles.

In this study, a 7 kg top-loading washing machine with a 54-L container was used to examine the factors that contribute to MF emissions. Each experiment involved using either 0.5 kg, 1.0 kg, or 1.5 kg of textile samples, 43 L of water, and the recommended dosage of commercial powder detergent, which was provided by the merchants at concentrations of 0.35 g/L, 0.70 g/L, and 1.05 g/L as shown in Figure 2.

The washing machine has 8 different washing modes: “Wash”, “Normal”, “Rinse”, “Soak”, “Spin”, “Delicate”, “Tub dry”, and “Quick”. For this study, the “Normal” washing mode was selected, which includes a 15-min washing step, followed by a 10-min rinsing step, and a 3-min draining step. After completing the washing step, the washing effluent was collected in a container, and then filtered for further analysis.

#### 2.2.2. Draining of Washed Water and Microfiber Collection Step

After the washing stage, the washing effluent was collected during each draining phase. The washed water was collected into a 10-L container using equally timed durations. To ensure homogeneous distribution of the released MFs, the collected washing effluent was continuously stirred. A 1-L sample of the washing effluent was then collected using a filtration system. The filtration process involved using a vacuum pump to draw the washing effluent through a 47 mm diameter filter paper with a pore size of 2.7 µm (Whatman GF/D glass microfiber filters, Hangzhou, China), as suggested by Cai et al. [45] and Wang et al. [46]. The choice of a 1 L filtration volume was to minimize the presence of overlapping MFs on the filters [47]. After filtration, all the filters were individually placed in petri dishes, covered with caps, and dried at 40 °C in an oven for 24 h. To prevent contamination from previous experiments, all laboratory materials employed in this study underwent a meticulous triple rinsing procedure with clean water before starting subsequent experiments. This rigorous and consistent process was implemented to prevent MF accumulation in the laboratory equipment.

### 2.3. Fourier Transform Infrared Micro-Spectroscopy (µ-FTIR) Analysis

**Figure 3:**
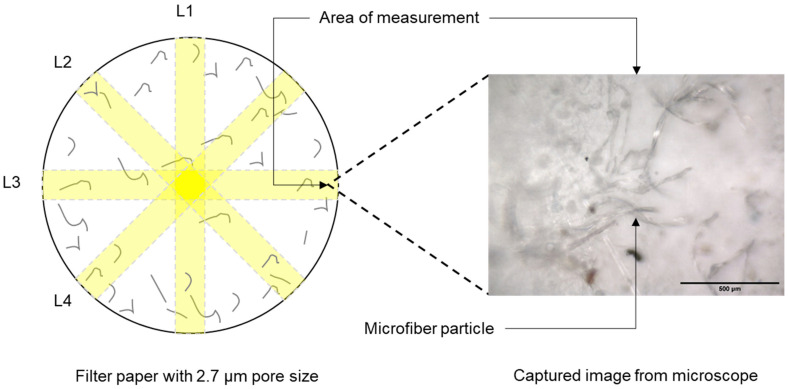
A schematic diagram illustrates how MFs were quantified using a microscope.

MFs collected by filtration were characterized using quantitative methods. To determine the number of MFs, multiple photomicrographs of the filter paper from each experiment were taken using a µ-FTIR (LUMOS II; Bruker Optics Inc., Ettlingen, Germany). These images were taken following the four directional lines (L1, L2, L3, and L4) as illustrated in Figure 3. MFs with dimensions of 1.50 mm × 1.42 mm that appeared in all the microscope-captured images (totaling 150–160 representative microphotographs) were manually counted. Finally, the number of MFs obtained in each image were used to calculate the concentration of released MFs in terms of particles/L.

## 3. Results and Discussion

### 3.1. Effect of Textile Weight and Detergent Dosage on MF Emissions

**Figure 4:**
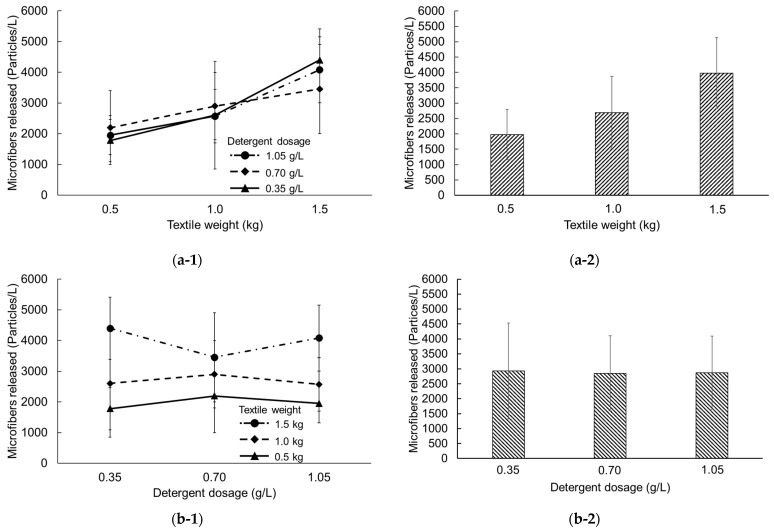
Microfiber emissions as a function of textile weight and detergent dosage: (**a-1**) MF emissions on textile weight in different detergent dosage, (**a-2**) arithmetic mean of all parameters that are equal in textile weight, (**b-1**) MF emissions on detergent dosage in different textile weight, and (**b-2**) arithmetic mean of all parameters that are equal in detergent dosage.

The amounts of MFs released from the textile washing experiments under various conditions are summarized in Tables S1 and S2 while the influence of textile weight and detergent dosage on MF emissions are presented in Figure 4. The observation revealed that washing 0.5 kg of textile with different detergent dosages (0.35, 0.70, and 1.05 g/L) yielded approximately 1780, 2196, and 1950 particles/L of MF emissions, respectively (Figure 4(a-1)), with the arithmetic mean depicted as 1975 particles/L in Figure 4(a-2). In addition, when the weight of the textile increased to 1.0 and 1.5 kg, the measured concentrations of MFs in the term of arithmetic mean were 2692 and 3978 particles/L, respectively. This suggests that there is a direct relationship between the concentration of MFs released in washing machine and the weight of the textile, with higher textile weight resulting in higher MF emissions. 

In contrast, detergent dosage did not significantly impact the concentration of released MFs. As illustrated in Figure 4(b-1), considering an equal textile weight of 1.0 kg, the results indicate that MF emissions were 2604, 2900, and 2573 particles/L when the detergent dosage increased from 0.35, 0.70, to 1.05 g/L. A fluctuating trend was observed in the relationship between MF emissions and detergent dosage, reflected in the arithmetic mean evaluation as depicted in Figure 4(b-2); MF concentrations for detergent dosages of 0.35, 0.70, and 1.05 g/L were 2927, 2850, and 2868 particles/L, respectively. These findings are inconsistent with a previous study by Wang et al. [46], which found that the addition of less than 1.5 g/L of detergent increased the emissions of MFs. This could be attributed to the presence of hard, inorganic compounds and natural minerals like zeolite, which are added to powder detergents as fillers and scrubbers. Because these materials are insoluble in water, they create friction with textile surfaces during laundry cleaning, improving cleaning efficiency but also promoting MF generation [35]. Lastly, it should be noted that the results found in this study are relative results, not absolute results. Although the results revealed the relationship of textile weight and detergent dosage on MF emissions, they may be an overestimate due to the use of scissors for sample preparation without stitching [48]. 

### 3.2. Effect of Textile Type on MFs Released in the Laundry Process

**Figure 5:**
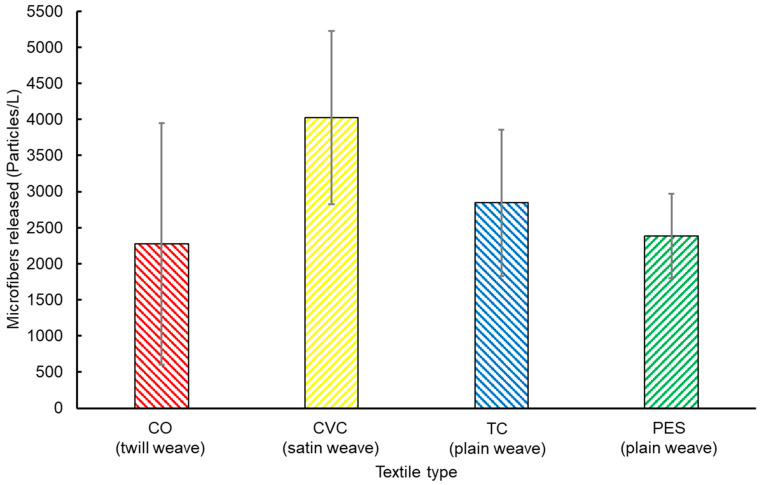
Microfiber emissions as a function of textile type during laundry cleaning in washing machines.

Figure 5 illustrates significant variations in MF emissions depending on the type of textile. The average MF concentrations released were as follows: 2279 particles/L for 100% CO, 4022 particles/L for CVC, 2844 particles/L for TC, and 2382 particles/L for 100% PES. It is worth noting that textiles containing synthetic fiber PES (such as 100% PES, TC, and CVC) had higher MF emissions compared to 100% CO. The higher MF generated by PES and PES-blended textiles could be attributed to the inherent hydrophobic nature of PES, which is a common property of plastics that makes them degrade more easily in the presence of surfactants found in detergents during laundry washing [49,50,51,52]. Additionally, both CVC and TC, which are PES-blended textiles, exhibited significant MF release, exceeding 76% and 25% of the amount of microfiber released by 100% CO, respectively.

### 3.3. Discussion on the Relationship of Woven Structure and Textile Type on Emission of MFs 

**Figure 6:**
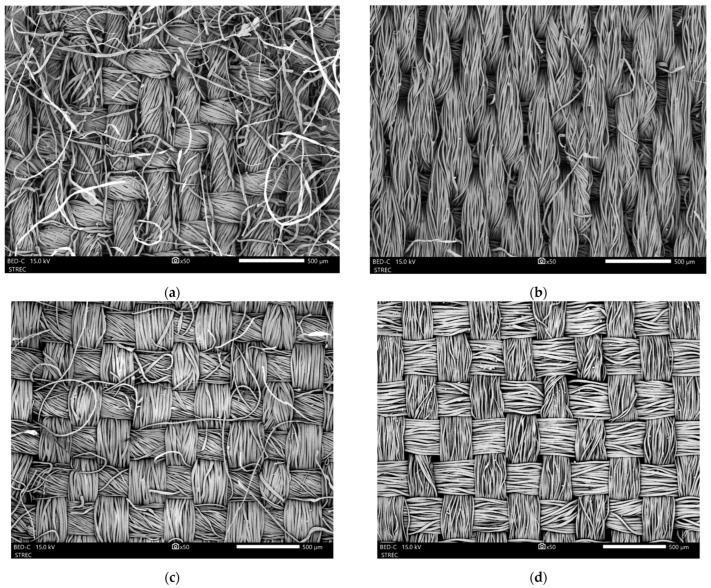
SEM photomicrographs taken at 15 kV (50× magnification) of the four textile types: (**a**) 100% CO; (**b**) CVC; (**c**) TC; (**d**) 100% PES.

To analyze the surface characteristics of 100% CO, CVC, TC, and 100% PES, observations were performed using a scanning electron microscope (SEM; JEOL JSM-IT300LV, JEOL Ltd., Tokyo, Japan). As illustrated in Figure 6, 100% CO had a twill weave structure (Figure 6a) while CVC had a satin weave structure (Figure 6b). Meanwhile, both TC and 100% PES had plain weave structures, as depicted in Figure 6c,d, respectively. 

**Figure 7:**
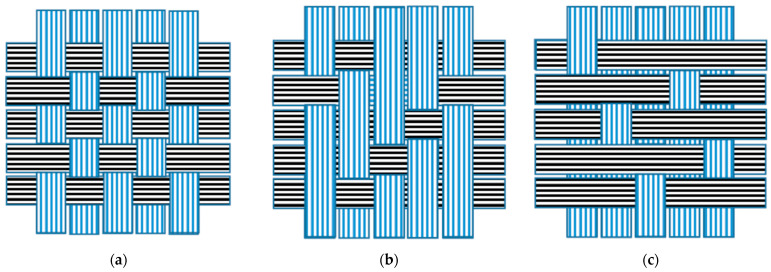
Schematic illustrations of the three common woven structures: (**a**) plain weave, (**b**) twill weave, and (**c**) satin weave.

Weaving is the process of intricately interlacing one-dimensional yarns to create two-dimensional and occasionally three-dimensional structures, such as woven fabrics or textiles. These fabrics have traditionally been used in clothing and apparel. In recent years, however, weaving technology has advanced, allowing for the customization of woven fabrics to meet specific performance requirements and technical applications. This is made possible by the versatility of fabrics and automation of weaving technology, which enables the use of various raw materials, including synthetic fibers like PES, to create products of different geometrical forms. The woven structure of textiles plays a crucial role in the generation of MFs during laundry washing because it directly affects the mechanical properties of textiles, such as the exposed surface area of fibers to water, detergent, and scrubbing/attrition action in washing machines. Figure 7 provides a schematic illustration of common woven structures in textiles, including the plain weave structure (Figure 7a), the twill weave structure (Figure 7b), and the satin weave structure (Figure 7c) [53,54]. 

Based on the results, the weave structure had a significant impact on the generation of MFs. This is to be expected because the mechanical properties of textiles, particularly their tensile strength and bending strength, influence how the fabric reacts to attrition and scrubbing forces during laundry cleaning in washing machines. Ferdous et al. [55] conducted experiments to measure the tensile and bending strengths of different woven structures and found that the twill weave structure exhibited the highest tensile strength, followed by plain weave and satin weave structures. Textiles with lower tensile and bending strength, such as CVC (satin weave), had the highest MF emissions, followed by PES and TC, both with plain weave structures. Meanwhile, CO, with a twill weave structure and the highest tensile and bending strengths, exhibited the lowest MFs emissions. This suggests that woven structures with higher tensile strengths result in lower MF generation. These differences could be explained by the way the threads are held in the different weave structures. In the twill weave and satin structures, the threads are not tightly held, allowing for better and faster dispersion of stress and strains across a greater number of yarns, which prevents damage and breakage. In contrast, the fibers in the plain weave structure are tightly held, limiting the dispersion of stress and strain. Additionally, the satin weave structure contains larger floats, which decrease its tensile and bending strength and promote mechanical degradation during laundry cleaning in washing machines.

The findings from this study emphasize the importance of conducting further research to better understand the mechanisms behind the increasing release of MFs from various types of textiles. Additionally, it is crucial to differentiate between MPFs and MFs from natural materials, such as CO, in order to gain a better understanding of how MPFs are released during laundry cleaning in washing machines. This classification will greatly contribute to our knowledge of the sources and characteristics of MPF pollution originating from domestic laundry washing.

## 4. Conclusions

In this study, the release of MFs during domestic laundry washing was investigated under various washing conditions, textile types and detergent dosages. The results showed that the concentrations of MFs varied depending on the type of textile. Specifically, textiles made of 100% CO exhibited the lowest release of MFs, followed by textiles made of 100% PES, TC, and CVC. The notably higher emissions of MFs from textiles made of 100% PES and blended fabrics containing PES could be attributed to the hydrophobic properties of this plastic-derived fiber, which enhanced its degradation due to the attachment of organic surfactants found in detergents used during laundry washing. Additionally, our findings revealed substantial release of MFs from textiles composed of blended natural and synthetic fibers like CVC, exceeding the release observed in 100% PES. This highlights the complexity of MF emission mechanisms and the need for further research to better understand MF generation and how it can be mitigated. Furthermore, the results showed that the dosage of detergent had negligible effects on the generation of MFs from all four types of textiles during laundry cleaning in washing machines. Finally, the woven structure of textiles was found to be another important factor that inherently influences the generation of MFs during laundry cleaning in washing machines. The results suggest that textiles with the twill weave pattern (100% CO) exhibited lower emissions of MFs due to their higher tensile and bending strengths compared to other woven structures like plain weave (100% PES and TC) and satin weave (CVC). 

## Data Availability

Data is contained within the article and Supplementary Materials.

## Supplementary Materials

The following supporting information can be downloaded at: https://www.mdpi.com/article/10.3390/toxics12030210/s1, Table S1: The summarized of experimental results based on the effects of textile weight on MF emissions; Table S2: The summarized of experimental results based on the effects of detergent dosage on MF emissions.
