# Supplementary material for: The Influence of Textile Type, Textile Weight, and Detergent Dosage on Microfiber Emissions from Top-Loading Washing Machines
Source: Toxics. 2024 Mar 12;12(3):210. doi: 10.3390/toxics12030210 (PMC10975110; doi:10.3390/toxics12030210)
Supplement: Supplementary file 1 [file toxics-12-00210-s001.zip › toxics-2853207-supplementary.pdf]

Supplementary materials

Table S1. The summarized of experimental results based on the effects of textile weight on MF emissions

Textile weight (kg)	Detergent dosage (g/L)	CO (Particles/L)	CVC (Particles/L)	TC (Particles/L)	PES (Particles/L)	Average	Whole average
0.5	0.35	758	2230	2003	2127	1780	1975
	0.70	529	3350	2240	2664	2196	
	1.05	2063	2643	1099	1996	1950	
1.0	0.35	397	3712	4275	2030	2604	2692
	0.70	1959	4134	3507	1999	2900	
	1.05	2135	3880	2207	2070	2573	
1.5	0.35	5146	5363	3785	3300	4399	3978
	0.70	3198	5397	3345	1878	3455	
	1.05	4326	5490	3136	3372	4081	

Table S2. The summarized of experimental results based on the effects of detergent dosage on MF emissions

Detergen t dosage (g/L)	Textile weight (kg)	CO (Particles/L)	CVC (Particles/L)	TC (Particles/L)	PES (Particles/L)	Average	Whole average
0.35	0.5	758	2230	2003	2127	1780	2927
	1.0	397	3712	4275	2030	2604	
	1.5	5146	5363	3785	3300	4399	
0.70	0.5	529	3350	2240	2664	2196	2850
	1.0	1959	4134	3507	1999	2900	
	1.5	3198	5397	3345	1878	3455	
1.05	0.5	2063	2643	1099	1996	1950	2868
	1.0	2135	3880	2207	2070	2573	
	1.5	4326	5490	3136	3372	4081	
